# Cercospora leaf spot disease of sugar beet

**DOI:** 10.1080/15592324.2023.2214765

**Published:** 2023-05-20

**Authors:** Wenbo Tan, Kexuan Li, Dali Liu, Wang Xing

**Affiliations:** a National Beet Medium-term Gene Bank, Heilongjiang University, Harbin, P.R. China; b Key Laboratory of Sugar Beet Genetics and Breeding, College of Advanced Agriculture and Ecological Environment, Heilongjiang University, Harbin, P.R. China

**Keywords:** Sugar beet, leaf spot disease, *Cercospora beticola*, epidemic, disease management

## Abstract

Leaf spot disease caused by *Cercospora beticola* Sacc. is the most damaging foliar disease threatening sugar beet production worldwide. The wide spread of disease incurs a reduction of yield and economic losses. The in-depth knowledge of disease epidemiology and virulence factor of pathogen is crucial and basic for preventing fungal disease. The integrated control strategies are needed for an efficient and sustainable disease management. The rotation of fungicides and crop could reduce the initial inoculum and delay the emergence of resistant pathogens. Spraying fungicides under the guide of forecasting models and molecular detecting techniques may hinder the onset of disease prevalence. The resistant varieties of sugar beet to cercospora leaf spot could be obtained by combining classical and molecular breeding methods. More effective approaches are supposed to develop for prevention and control for fungal disease of sugar beet.

## 1. Introduction

Sugar beet (*Beta vulgaris* L.) domesticated from the sea beet (*Beta maritima* L.) has been worldwide cultivated in more than 40 countries.^1^ It is the second most cultivated sugar crop after sugarcane and provides about 20% of the global sugar production.^2^ Cercospora leaf spot (CLS) caused by *C. beticola* is one of the destructive plant diseases of sugar beet. It reduces the quality of sugar beet and results in a loss exceeding 30% of the yield. To mitigate the impact of CLS on sugar beet, much progress in understanding the pathogen and its epidemiology has been made by global researchers. The epidemic and pathogenic mechanism of *C. beticola*, genetical resistance research and disease management were reviewed to summarize the strategies for CLS disease prevention and control.

## 2. Epidemic of cercospora leaf spot disease

The genus *Cercospora* was first described by Saccardo in Italy.^3^ In 1886, the pathogen distribution map, spread and pathogenicity, as well as disease symptoms of CLS were initially documented.^4^ Several decades after its initial description, the global dissemination of the CLS pathogen emerged as a significant challenge for sugar beet cultivation. According to a recent survey on the distribution of CLS, more than one-third of the global sugar beet area is affected by it. The United States, Austria, Greece, Italy, Hungary, etc. have a high incidence of the disease.^5^

**Figure 1.**
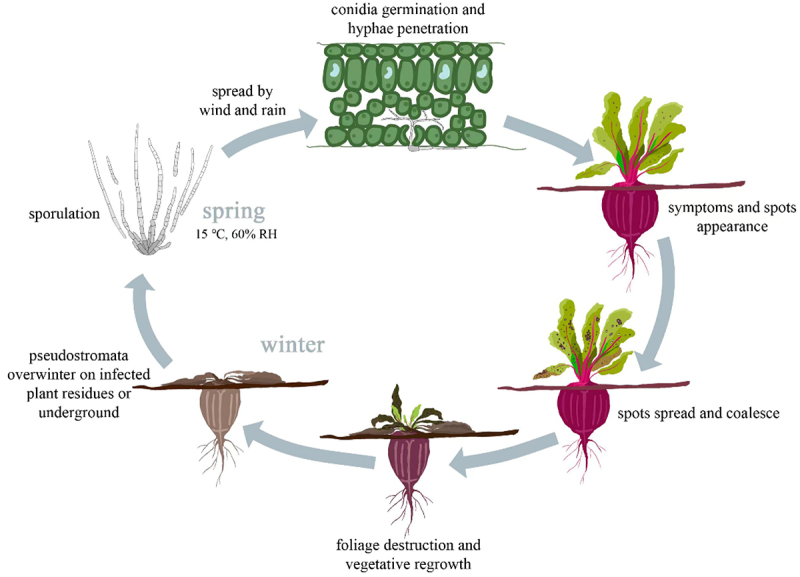
Life cycle of cercospora leaf spot disease of beets.

*Cercospora beticola* overwinters underground on infected plant residues in the form of pseudostroma. Dry environments are conducive to survival of pathogens. When temperature and humidity levels become suitable in the spring, the conidia begin to generate and invade plants through rain, wind, and insects.^6^ The abaxial leaf surface and petiole of sugar beet are the primary infection sites. The process of infection initiates with spore germination and then germ tubes penetrate the stomata. After appressorium formation, hyphae spread in mesophyll tissue and then produce toxins,^7,8^ which cause cell necrosis and leaf spots (Figure 1).

The spread of conidia mainly occurs in a limited range of agricultural field.^9^ However, long-distance asexual spread can also occur through machinery, insects, water flow, and other factors. Strains of *C. beticola* collected from different fields show low level of population differentiation and high genotype duplication.^10^ The trade of sugar beet also provides an opportunity for further spread of pathogen across different continents.^11^ During the quality inspection of sugar beet seeds, *C. beticola* is often detected to exist in the seed coat and then induce CLS along with sowing.^12^

## 3. Virulence factors of *C. beticola*

Cercosporin is a photoactive toxin, which was first isolated by Kuyama in 1957.^13^ Its toxicity in mice was related to the intensity of light.^14^ Cercosporin exists widely in the fungi of genus *Cercospora*. It could stimulate the generation of reactive oxygen species (ROS) in organisms. When ROS was excessive, cell structure collapse was caused by membrane lipid peroxidation and electrolyte leakage.^15^ The first cercosporin biosynthesis gene *CTB1* was found in the mutant of *C. nicotianae*.^16^ *CbCTB2* encodes a *O*-methyltransferase, of which the mutant failed to produce cercosporin and colonize sugar beet leaves.^17^ It proves that cercosporin is not only involved in the initial penetration and colonization but also plays a role in biotrophic phase of the pathogen. Genome sequencing indicated *C. beticola* held 63 secondary metabolic gene clusters. The *CTB* gene cluster had taken multiple duplications and horizontal transfer to other pathogenic fungi.^18^

Beticolin is a non-host-specific phytotoxin. This yellow compound was first named in studying the effect of toxins of *C. beticola* on cell membrane.^19^ Twenty beticolin compounds have been found from *C. beticola* but the biosynthetic pathways and toxicity were still unclear.^20,21^ In addition to cercosporin and beticolins, there are some potential virulence factors, including cellulases, pectinases and melanin.^22^ In 2021, Ebert et al. found that *C. beticola* secreted an effector protein CbNip1 that could penetrate the sugar beet leaves and lead to necrosis. Darkness is essential for full CbNip1-triggered cell necrosis. Infiltration of CbNip1 protein into sugar beet leaves showed that host cell death occurred more rapidly and extensively under dark conditions compared to light conditions.^23^ It complements photoactivated necrosis formation by cercosporin. The expression of plant pathogenesis-related genes is usually inhibited by darkness, which is benefit for effector proteins to produce toxicity in leaf cells.^24^ The study on CbNip1 is helpful to illustrate the plant–pathogen interaction process in darkness and further understand the occurrence of CLS.

## 4. Disease management

The symptom development of CLS on sugar beet crop is a gradual progress. In the first stage, necrotic spots emerge on the primary leaves. As CLS disease progresses, the primary foliage of the plant starts to wilt and fall off. This can lead to a vegetative regrowth to maintain photosynthetic capacity. However, this regrowth is stimulated at the expense of sugar reserves in root. It results in a significant loss of root weight and sugar content, so the control measures for CLS are very necessary for the field management.^25,26^

### 4.1 Cropping system

Crop rotation is an important method for reducing the amount of plant pathogens in field. Rotation with non-host crop is a practical strategy for disease management that could reduce initial inoculum load in the next year. At least 2-y rotation is necessary to reduce the levels of initial infection, as shorter rotations may still allow for significant survival of fungal pathogens and result in greater disease prevalence and intensity in sugar beet crops.^27,28^ In addition, crop rotation can also help improve soil fertility and structure, reduce soil erosion, and manage weeds or pests. Deep tillage may further relieve initial inoculum pressure in the next season. Because spores can disperse long-distance by wind, the sugar beet should be planted far away from the previously infested fields.

### 4.2 Assessment and monitoring

Image analysis is a technology that collects the images of sugar beet leaves with either a camera or other imaging tools. Software such as Scion Image and MATLAB can then be used to analyze the data.^29^ Artificial image collection holds the disadvantages of low efficiency and high error rate, while unmanned aerial vehicles (UAVs) and unmanned ground vehicles (UGVs) are high throughput vectors that overcome the defect in fieldwork. Although UAVs are limited to illumination and wind conditions, they could supply the images of high spatial resolution. UGVs can provide high-resolution images to identify the CLS symptoms by short-distance active sensors, but they require strict soil conditions in practical operation.^30^ ASSESS is a piece of image analysis software that identifies the necrosis region of leaves. It calculates the area by pixels which avoids overestimation of disease severity by visual assessment.^31,32^ In recent years, deep learning and artificial intelligence technology have offered great convenience to agricultural production. Convolutional neural network (CNN) is a feed-forward neural network constructed by imitating biological visual perception, which holds powerful capabilities in the field of image processing.^33^ The regression model developed by combining CNN with dataset can process images with the fewest errors.^34,35^

Polymerase chain reaction (PCR) is highly specific and sensitive, which could be applied for identifying the potential risks of CLS prevalence. It detects the unique DNA fragments of *C. beticola* from asymptomatic leaves, alternate hosts, and seeds, even spores from artificial traps. It is particularly useful in the initial stage of disease diagnosis and in the prevention of CLS spread.^36,37^ The enzyme-linked immunosorbent assay (ELISA) is an antigen-based detection method. By combining ELISA with PCR technology, it could not only screen the pathogens in soil quickly but also quantify the mycelial biomass sensitively.^38,39^

### 4.3 Chemical and biological treatments

Fungicide is the most effective tool employed in CLS disease prevention. At present, a series of protective and curative fungicides such as dithiocarbamates, benzimidazoles and amines are used by growers for disease control.^40^ Protective fungicides are locally applied to hinder the infection of conidia, while curative fungicides can be transported between different plant organs. The alternation of different fungicides is encouraged to avoiding the acceleration of resistant strains.^41^ When the mineral elements (e.g., CuSO_4_, S and H_3_BO_3_) were sprayed mixed with fungicides, the activity of catalase, peroxidase and polyphenolosxidase of sugar beet increased significantly that could ease the damage of plant cells.^42^

Biological control is an alternative approach that shows the potential for CLS prevention. *Trichoderma* strains could not only inhibit the growth and sporulation of fungi in vitro, but also cut down the overwintering conidia of *C. beticola*.^43^ After inoculating sugar beet with the strains of *Bacillus*, *Emericella* and *Epicoccum*, the severity of CLS was reduced and the activity of chitinase and glucanase all increased. The pathogen-resistant proteins chitinase and glucanase are recognized as two molecular markers associated with pathogen-induced systemic acquired resistance. They have a synergistic effect leading to fungal pathogen control, which is not evident when either occurs independently. The enzymes might reduce fungal disease severity by degrading the chitin and glucan components of fungal cell walls.^44,45^ Additionally, the isolates of *Paenibacillus* and *Pseudomonas* could be used as biocontrol agents for CLS of beet. The activities of peroxidase, polyphenoloxidase and phenylalanine ammonia lyase in beet plants increased significantly after treatment with them.^46^ Phenylalanine ammonia lyase initiates the phenylpropanoid pathway, which leads to the production of phytoalexins and phenolics. Polyphenoloxidase and peroxidase could induce lignin biosynthesis and oxidative cross-linking of plant cell walls, which can help prevent the spread of pathogens.^47,48^

### 4.4 Resistant varieties

Endowing sugar beet varieties with genetic resistance is effective for limiting CLS in field. A series of defensive responses initiated by the related genes expression is responsible for delaying the infection process and disease development.^49^ As a wild ancestor of sugar beet, sea beet is often used as a source of resistant genes in breeding programs, in which the backcrossing method is often applied.^50^ Resistance to CLS is inherited in a quantitative mode and 4–5 genes are estimated to be involved.^51^ These quantitative trait loci (QTLs) that are mostly partially dominant or additive distribute on different chromosomes. The heredity is affected by environmental factors.^52^ The broad and narrow sense heritabilities for resistance are 70% and 25%, respectively, and the environmental variation range from 44% to 62%.^53^

In order to select the genotypes of sugar beet that are highly resistant to CLS, the biochemical-molecular substances such as peroxidase, polyphenol oxidase and chitinase are often used as biomarkers.^54^ Meanwhile, the abundance of bacterial endophytes such as *Methylobacterium* and *Mucilaginibacter* can be correlated with increased sensitivity to CLS disease.^55^ These biomarkers could be employed as indicators for breeding resistant varieties of sugar beet. Compared with the analysis of differential proteins expression, metabolites profile is a more convenient method in practice. 4-Aminobutyrate, fructose and glutamine could differentiate the susceptibility of genotypes.^56^ In addition, fungitoxic saponins and polyphenolic compounds are associated with higher host resistance.^57,58^

### 4.5 Integrated management

Varieties of sugar beet have been developed with genetic resistance to CLS, and these varieties have been used successfully in many parts of the world where sugar beet cultivation faces challenges with this disease. However, the negative association between disease resistance and sugar yield can make it difficult to develop sugar beet varieties that combine both high levels of disease resistance and high productiveness.^59^ When disease pressure is low or absent, very productive but susceptible sugar beet varieties that receive a modest number of fungicide treatments may perform better than resistant varieties because they have better inherent yield potential.^60^ When CLS are prevalent and heavy epidemics occur frequently, economic yields typically require the combined use of resistant varieties and repeated fungicide treatments.^61^ However, it is important to take into account the drawbacks of fungicides, including potential negative effects on human health and the environment, increased production costs, and the development of fungicide-resistant pathogen strains. Field monitoring and epidemic forecasting models are more efficient integrated approaches that could minimize the number of fungicidal treatments. By providing early warning of disease onset and progression, the models allow growers to schedule fungicide treatments more effectively and prevent unnecessary applications. Some models based on weather data calculate the cumulative daily infection value of CLS and predict the infection process.^62^ Other models take into account the disease incidence, agronomic characteristics and weather data, which assist disease management comprehensively.^63,64^

## 5. Future prospects

As mentioned above, enormous progress has been made to manage the cercospora leaf spot disease, which continues to be a serious problem for sugar beet. The advanced and integrated approaches are in great need for production. Spraying fungicides should be with the mode of alternation to reduce the risk of pathogen resistance. Comprehensive models are needed for more precise prediction of disease, which could reduce the use of fungicide.

Molecular methods are useful for detecting the pathogen at the early stage of disease prevalence. It also could determine alternative and asymptomatic hosts that serve as pathogen reservoirs.^49^ The technologies for biological control of CLS are being deployed. In addition to the microbes that could be used as antagonist to *C. beticola*, some classes of enzyme isolated from them might detoxify cercosporin.^65,66^ The plant diseases vaccine such as oligochitosan, which is effective at eliciting plant innate immunity against plant diseases, is a fascinating research direction.^67^

The transgenic sugar beet has significant potential and commercial value for CLS control. The resistance can be acquired by the expression of transgenes that encode detoxifying enzymes, anti-fungal peptides and phytoalexins.^68,69^ Gene editing has developed rapidly for crop improvement in recent years. When it is adopted for resistance breeding, the targets are often genes that negatively regulate the anti-disease responses. It also aims to disease responses including the proteins encoding genes that are hijacked by pathogens for infection. To achieve the success that has been attained in wheat resistance breeding for powdery mildew, gene editing techniques will be applied on sugar beet soon.^70^
